# The Value of Neutrophil-to-Lymphocyte Ratio to Identify Bacterial Infection and Predict Short-Term Mortality in Patients with Acutely Decompensated Cirrhosis

**DOI:** 10.3390/diagnostics13182954

**Published:** 2023-09-14

**Authors:** Tamás Janka, Dávid Tornai, Mária Papp, Zsuzsanna Vitális

**Affiliations:** 1 Division of Gastroenterology, Department of Internal Medicine, Faculty of Medicine, University of Debrecen, 4032 Debrecen, Hungary; janka.tamas@med.unideb.hu (T.J.);; 2 Kálmán Laki Doctoral School of Biomedical and Clinical Sciences, University of Debrecen, 4032 Debrecen, Hungary

**Keywords:** liver cirrhosis, mortality, acute decompensation, neutrophil-to-lymphocyte ratio, C-reactive protein, procalcitonin, white blood cell, infection

## Abstract

Liver cirrhosis patients are highly susceptible to infections, affecting survival, but current parameters for detecting infection are not reliable enough in this population. We investigated the ability of white blood cell (WBC), ∆WBC, neutrophil and ∆neutrophil counts, neutrophil-to-lymphocyte (NLR) and ∆NLR ratios and C-reactive protein (CRP) and procalcitonin (PCT) levels to identify infection and predict short-term mortality in liver cirrhosis patients. We recruited 233 patients with liver cirrhosis hospitalized with acute decompensation (AD) who had an outpatient visit within 1 month (baseline laboratory data) and followed them for 90 days. Difference between laboratory values at baseline and the AD episode was defined as delta (∆) values of the parameters. Delta values did not increase the diagnostic and predictive ability of investigated parameters. The CRP level was found to be the best diagnostic marker for infection in patients with cirrhosis. However, NLR seems to be superior for short-term mortality prediction, better than the WBC count. Distinguishing inflammations of different origin is a remaining clinical challenge in acutely decompensated cirrhosis. Based on our results, NLR might be more suitable for predicting short-term mortality in patients with AD than the WBC count currently included in the CLIF-C AD score.

## 1. Introduction

Patients with liver cirrhosis have high susceptibility to infection. Bacterial infections affect not only short-term [1] but also long-term survival [2]. Their timely detection may improve the outcome, but the routinely used parameters that indicate infection are not sufficiently effective in liver cirrhosis [3]. 

In liver cirrhosis, pathogenic bacterial translocation may result in a significant bacterial burden (pathogen-associated molecular patterns—PAMPs), which together with danger-associated molecular patterns (DAMPs) due to liver (and other organ) damage may activate the immune system without a manifest infection, resulting in persistent inflammation depending on the stage of cirrhosis. This may elevate the levels of inflammatory markers [4] even in the absence of an overt infection making their use less reliable compared to the noncirrhotic population. Moreover, C-reactive protein (CRP), the most widely used marker for screening inflammation, is produced by the liver; thus, the CRP response to inflammation could be slow and inadequate as the liver cirrhosis worsens due to the deterioration of liver function. Procalcitonin (PCT) [5] is not a fully reliable marker either, as the associated renal failure is common in advanced stages of liver cirrhosis that may increase the PCT level even in the absence of infection. In patients with hypersplenia-derived leukopenia, the white blood cell (WBC) count may be within the normal range, even at times of an incipient leukocytosis. Furthermore, symptoms are also atypical in about half of the cases [3]. 

Due to the increasing antibiotic resistance of bacteria, antibiotic use needs careful consideration. In the case of an infection, an immediate effective antibiotic treatment is essential; however, when inflammation is not due to infection, antibiotics should be avoided. Therefore, there is a great need to have a marker at hand that is effective in detecting bacterial infections early in the process. 

Since 1999, it has been known that a bacterial lipopolysaccharide in the circulation causes a temporary decrease, followed by an increase, in neutrophil cell numbers within a few hours and a parallel decrease in lymphocyte numbers [6]. The neutrophil cell count indicates the activation of innate immunity, while the change in lymphocyte count is related to the activation of the adaptive immune response [7]; therefore, the neutrophil-to-lymphocyte ratio (NLR) characterizes the balance between the initial innate and adaptive immune responses. However, it seems that the normal value of NLR is not easy to determine. It depends on race and lifestyle-related risk factors, such as smoking or excess weight [8]. The mean value may be somewhere around 1–2 [9]. Certain diseases, such as diabetes mellitus, cancer, atherosclerosis or ischemic heart disease, psychiatric disorders, subclinical infection and/or inflammation, are known to be associated with chronically elevated NLR. Low-grade inflammation just slightly elevates the level of NLR [7]; therefore, it might be helpful in differentiating between infection and sterile inflammation. 

In liver cirrhosis, NLR is expected to increase with disease progression, but there are insufficient data [10]. The value of NLR is influenced by ongoing infection, trauma, hemorrhage, etc., so, within a given Child–Turcotte–Pugh (CTP) stage, NLR is supposed to be higher in the presence of infection and associated with worse survival [11,12]. 

In our study, we sought to answer the above challenges by comparing the CRP level, WBC count, neutrophil count and NLR value measured at admission for an acute decompensation (AD) event. We also hypothesized that using the patients’ own values measured during a regular outpatient visit (non-AD) as reference rather than comparison with values from the healthy population would increase diagnostic accuracy.

## 2. Materials and Methods

### 2.1. Patient Population 

We conducted an observational cohort study of 233 adult patients with established diagnosis of cirrhosis of different etiologies in a referral hepatology center (Division of Gastroenterology, Department of Internal Medicine, Clinical Center, University of Debrecen, Hungary). 

Between 1 December 2016 and 30 September 2020, 736 patients with cirrhosis were screened. Of these patients, 233 were selected who had an acute decompensation event at hospital admission and had an outpatient visit within 1 month before this episode. The results of the outpatient visit were considered as baseline values. The exclusion criteria were as follows: (1) the patient or legal surrogate declined to participate in this study and did not sign the informed consent, (2) the patient was sent for a single specialist consultation only and was followed up regularly elsewhere, or (3) the patient had no complete blood count at admission for acute decompensation or baseline blood count available.

The diagnosis of cirrhosis was based on clinical, biochemical, imaging and, when available, histological data as described previously [13]. Routine laboratory data and detailed clinical phenotypes were captured at inclusion. Clinical data were determined by the in-depth review of detailed medical records (age at diagnosis, etiology, presence of hepatocellular carcinoma (HCC)). Outpatient data prior to the hospital admission were retrospectively collected and analyzed. Disease severity was assessed by liver-related scores: CTP [14,15] and model for end-stage liver disease (MELD) [16,17]. At acute decompensation, CLIF-C AD score [18,19,20] was also calculated. ACLF (acute-on-chronic liver failure) [21] was diagnosed, and ACLF grade was determined according to the European Association for the Study of the Liver–Chronic Liver Failure Consortium (EASL-CLIF) criteria [21]. We divided the patients into three groups as follows: (1) CLIF-AD score <50 or (2) ≥50, or (3) the patient had ACLF. 

Infection was diagnosed on the basis of conventional criteria (clinical symptoms, appearance of fever and laboratory data, including microbiological culture results, if available, and compatible findings of imaging techniques) as detailed in our previous study [13]. Our patients had spontaneous bacterial peritonitis, airway infection, cholangitis, skin and soft tissue infection and urinary tract infection.

### 2.2. Routine Laboratory Markers of Inflammation

Samples were taken for routine laboratory analysis on the first day of admission and on the day of outpatient (baseline) visit. NLR was calculated by dividing the absolute neutrophil count by the absolute lymphocyte count, given by the differential white blood cell count. The alteration of NLR, neutrophil count and lymphocyte count were calculated by extracting the baseline values from the values measured at the time of a decompensation event (∆NLR, ∆neutrophil count, ∆lymphocyte count). CRP and PCT measurements were performed routinely only at time of AD. In some cases, patients had CRP (n = 192) but not PCT measurement at baseline visit. Routine laboratory tests were performed at the time of hospital admission and outpatient visit. 

### 2.3. Data Collection 

The data, comprising clinical variables, laboratory results and outcomes, were collected prospectively for the AD episode and retrospectively for the outpatient visit. 

Attending gastroenterologists registered the date and type of acute decompensation episode during the hospital admission of the patients in this study. Acute decompensation was defined by the acute development of large ascites (grade II/III), acute hepatic encephalopathy, acute variceal bleeding or the presence of systemic bacterial infection as detailed previously [22]. The follow-up period lasted 90 days or until death. Collected data were transferred to and stored in a database. At the end of the study period, 30 September 2020, attending gastroenterologists checked medical records registered during regular and extraordinary outpatient follow-up visits and inpatient stays to identify and gather baseline laboratory parameters.

### 2.4. Ethical Permission 

This study was conducted according to the guidelines of the Declaration of Helsinki and approved by the Regional and Institutional Research Ethics Committee of University of Debrecen and the National Scientific and Research Ethics Committee (protocol code DEOEC-RKEB/IKEB 5306–9/2011, 3885/2012/EKU [60/PI/2012], 9485-1/2016/EKU ad 167/2016).

### 2.5. Statistical Analysis 

Variables were tested for normality using Shapiro–Wilk W test. Continuous variables were summarized as means (SD) or as medians (interquartile range (IQR), lowest 25%–highest 25%) according to their homogeneity. Categorical variables were compared with Fisher’s exact test or χ2 test with Yates’s correction, as appropriate. Continuous variables were compared with Mann–Whitney U test or Kruskal–Wallis H test with Dunn’s multiple comparison post hoc analysis. Paired samples were analyzed by Wilcoxon signed-rank test. Ability of different variables to discriminate between patients with or without infection as well as survivors and nonsurvivors were assessed by receiver operating characteristic (ROC) curve analysis plotting sensitivity% vs. 100-specificity%. Area under the curve (AUROC) and corresponding 95% confidence intervals (CI) were calculated. Youden’s index was chosen, calculated as the maximum (sensitivity + specificity) value, to estimate the best discriminative threshold. The association between laboratory variables and the presence of infection was assessed by univariable logistic regression. Odds ratios (ORs) with 95% CIs were calculated. The association between laboratory variables and short-term mortality during follow-up was assessed by univariable Cox regression analysis. These associations are given as hazard ratios (HRs) with CIs. For statistical analysis and graphical presentation, the SPSS 29.0 (SPSS, Chicago, IL, USA) and GraphPad Prism 9.5.1 (San Diego, CA, USA) programs were used. A two-sided probability value of <0.05 was considered to be statistically significant. 

## 3. Results

### 3.1. Patient Characteristics at Inclusion (AD Episode) and Baseline

Of the 233 patients with acutely decompensated cirrhosis, 126 (54%) had bacterial infection as a predisposing factor causing the AD event. Table 1 summarizes the clinical and relevant laboratory characteristics of infected and noninfected patients. 

Baseline patient characteristics are detailed in Table 2.

### 3.2. Increased NLR Is Associated with AD and More Severe Disease 

**Figure 1:**
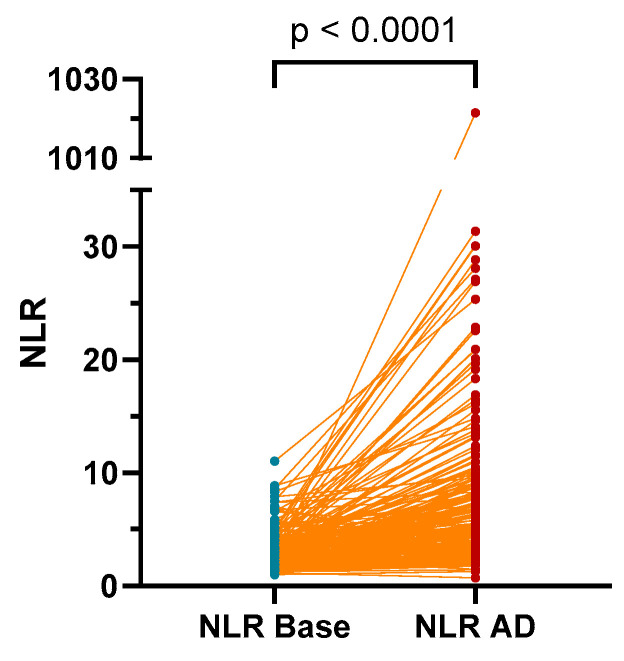
The NLR values were found to be significantly higher in patients during an AD event compared to values measured at AD-free outpatient visit (base). AD: acute decompensation; NLR: neutrophil-to-lymphocyte ratio.

The NLR values were found to be significantly higher in patients during an AD event (median (IQR): 2.76 (2.07–3.79)) compared to values measured at baseline (AD-free outpatient visit; 4.77 (3.025–8.035), *p* < 0.0001; Figure 1).

At baseline, we found no correlation between NLR and CTP score (*p* = 0.088) or MELD score (*p* = 0.791). However, during AD episodes, NLR (*p* = 0.022), WBC count (*p* = 0.005), CRP level (*p* = 0.001), neutrophil count and percentage (*p* = 0.002 and *p* < 0.001) showed significant correlation with the CTP score. Similar results were also observed in relation to the MELD score (NLR, WBC count, CRP level, neutrophil count and percentage: *p* < 0.001) (Table 3). 

**Figure 2:**
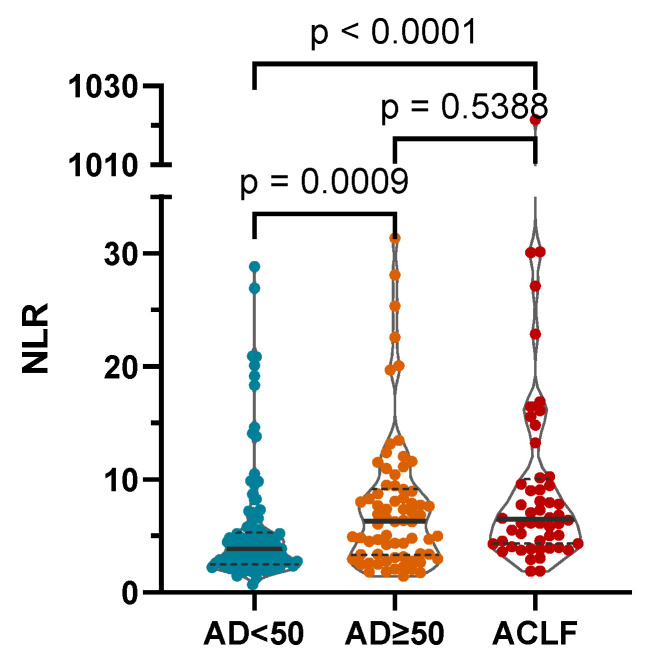
The NLR was significantly higher in more severe stages of AD as defined by the CLIF-C AD score and in the presence of acute-on-chronic liver failure (ACLF). AD: CLIF Consortium acute decompensation score; NLR: neutrophil-to-lymphocyte ratio; *p-*values of <0.05 are statistically significant.

The NLR was significantly higher in more severe stages of AD as defined by the AD score and in the presence of ACLF (median (IQR): CLIF-C AD score < 50: 3.88 (2.49–5.32); CLIF-C AD score ≥ 50: 6.33 (3.31–9.18); ACLF: 6.49 (4.32–10.01); Figure 2). 

### 3.3. WBC-Derived Parameters Are Moderate Indicators of Infection

Infection was associated with increased absolute number of neutrophils as well as neutrophil percentage, NLR, CRP and PCT levels. A greater increase in WBC and neutrophil counts as well as NLR compared to baseline was also significantly associated with infection (Table 1). Among these variables, the highest discriminative power as defined by the AUROC was found for the CRP level (0.736, *p* < 0.001) with a cutoff value of 15.48 mg/L (sensitivity: 76.19%; specificity: 65.09%). The AUROC for NLR (0.66, *p* < 0.001), ∆NLR (0.662, *p* < 0.001), PCT (0.688, *p* < 0.001) were in the moderate range. Notably, the best discriminatory cutoff value for NLR was 5.32 with a sensitivity of 57.76% and specificity of 72.9%. Furthermore, the AUROC for WBC count was clearly inferior to the above-mentioned parameters (0.568, *p* = 0.072; Table 4). However, in the univariable logistic regression, all the investigated parameters were associated with the presence of infection with moderate ORs (Table 4). 

### 3.4. CRP Identifies Infection with Superior Efficacy in Every Severity Group

**Figure 3:**
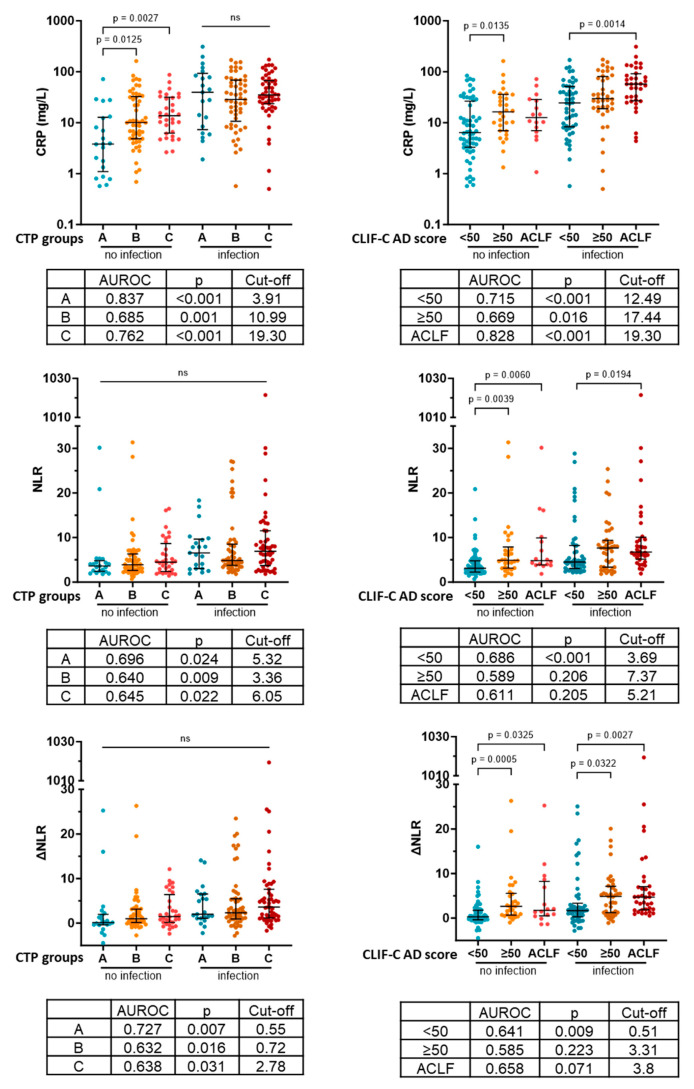
Levels and ability to identify infection of CRP, NLR and ∆NLR in different severity categories. CTP: Child–Turcotte–Pugh; CRP: C-reactive protein; CLIF-C AD score: CLIF Consortium acute decompensation score; ACLF: acute-on-chronic liver failure; AUROC: area under the receiver operating characteristic curve; NLR: neutrophil-to-lymphocyte ratio; ΔNLR: baseline NLR–NLR at acute decompensation; *p-*values of <0.05 are statistically significant.

Next, we investigated how the discriminative power of CRP changes in more severe disease and whether NLR can assist the identification of patients with infection in certain severity categories. We found CRP levels to be elevated in more severe disease groups of noninfective patients compared to the least severe disease group defined by both the CTP stage (A: 3.81 [1.10–12.74]; B: 10.05 [4.83–32.56]; C: 13.73 [6.24–31.53]) and AD score/ACLF (<50: 6.385 [3.29–26.43]; ≥50: 16.31 [6.95–36.01]; ACLF 12.59 [6.98–28.48]. This caused the best discriminative cutoff level to be gradually increased in more sever groups (Figure 3). NLR and ∆NLR did not show any CTP score-associated pattern. On the other hand, NLR and ∆NLR were elevated in patients with CLIF-C AD score ≥50 and ACLF compared to patients with CLIF-C AD score <50 both in the presence and absence of infection. Consistently the best discriminative cutoff values were also increased in these severity categories. However, the CRP level showed superior diagnostic value in all cases (Figure 3). 

### 3.5. Infection-Related Parameters Are Associated with Short-Term Mortality in AD Patients

In the ROC analysis, the WBC and ∆WBC counts performed poorly in identifying nonsurvivor patients both at day 28 (0.059, *p* = 0.099 and 0.601, *p* = 0.036) and day 90 (0.583, *p* = 0.057 and 0.59, *p* = 0.039) of the follow-up. The PCT level (0.748, *p* < 0.001 and 0.740, *p* < 0.001) had the highest AUROCs followed by NLR, ∆NLR and neutrophil percentage at both time points. The CRP level had slightly smaller AUROCs (Table 5). In the univariable Cox regression, the WBC and ∆WBC counts were found to be predictors of 90-day mortality but not 28-day mortality. All the other parameters predicted mortality at both time points with NLR having the highest HRs (HR: 1.772 (95% CI: 1.386–2.264), *p* < 0.001; HR: 1.830 (95% CI: 1.465–2.285), *p* < 0.001) (Table 5). 

### 3.6. NLR and ∆NLR Are the Best Predictors of Short-Term Mortality in Non-ACLF AD Patients 

Since WBC count is included in the CLIF-C AD score, we investigated predictive power of the WBC count, ∆WBC count, CRP, PCT, NLR and ∆NLR in non-ACLF AD patients. In the univariable Cox regression, only NLR and ∆NLR predicted both 28- and 90-day mortality. PCT was a predictor of 28-day mortality, but the WBC and ∆WBC counts and the CRP level were not significant in either time points (Table 6). 

## 4. Discussion

In our research, we sought to find out whether certain inflammation-related blood cell parameters could aid in improving the efficacy of early detection of infections provided by current gold-standard methods (CRP and PCT). The progression of liver cirrhosis triggers increasing and persistent inflammation in the body, and yet it is often accompanied with a lower basal WBC count in advanced stages. Thus, we hypothesized that it could be beneficial to consider the patient’s basal values (measured at an outpatient visit) as a reference instead the normal range in healthy individuals during the evaluation of AD. Therefore, we examined whether the absolute WBC count, its deviation from the basal value (**∆**WBC), the neutrophil count, the neutrophil percentage, the NLR or the **∆**NLR provide greater assistance in this regard.

In the absence of AD, the NLR value did not significantly differ depending on the disease severity (CTP, MELD score) in our patients. This result was unexpected, as the progression of the disease is associated with intensifying chronic inflammation in the body, and NLR is a ratio indicative of the extent of inflammation. A possible explanation could be the presence of hypersplenism in a significant number of patients, which may influence the proportions of white blood cell types. Contrary to our results, a previous study reported higher NLR values in patients with CTP C stage [23]. This discrepancy in results might be attributed to our separate analysis of cases with and without AD, whereas the previous study investigated a population including both non-AD and AD cases. Importantly, the latter group inevitably includes patients with more severe disease. However, in AD, we found the NLR to be significantly correlated with disease severity (CTP and MELD scores), which is in line with the results of Sun et al. [24] who reported that the NLR correlated with the CTP score in patients with ACLF. Furthermore, the NLR was also increased in more severe AD assessed by AD-specific scores (CLIF-C AD score and the presence of ACLF). Additionally, the NLR values were elevated during AD compared to an AD-free period in the same patients.

In our study, similar to the results of Kwon et al. [10], the NLR and CRP level were higher in patients admitted with infection-related AD compared to noninfectious AD patients. Using the best discriminatory cutoff value of 5.32 for identifying infections, the sensitivity of NLR was only 54.76%, which was significantly weaker compared to the sensitivity of the CRP level at a cutoff value of 15.48 mg/L (76.19%). Deutch et al. [5] using a slightly lower cutoff value (10 mg/L) found that the CRP level indicated infection with a sensitivity of 68%, a specificity of 84.55% and an AUROC of 0.8197. On the other hand, NLR proved to be more helpful in identifying infection than WBC or neutrophil count. In the ROC analysis, the CRP level had the highest area under the curve, while the logistic regression analysis revealed the highest odds ratio for PCT. NLR performed moderately well in both analyses but fell short compared to acute-phase proteins.

A recent meta-analysis [25] found no significant difference in cumulative NLR levels depending on whether or not patients had an infection, although almost all of the selected articles concluded that there was a significant difference between groups. The discrepancy was due to the marked variation in the NLR ranges found in each study. NLR levels are not only determined by infection, but are also influenced by other types of inflammation, comorbidities, body weight, but there are also differences between races [8]. It is unknown which of these parameters differed between the included articles, but it is clear that very distinct populations were evaluated in these studies. In addition, the selected articles included some that did not use the NLR to detect infection, but rather to predict the development of infections during hospitalization [26] or mortality [27]. One of the cited articles also confused diagnosis and prediction by assessing community- and hospital-acquired infections together, while the NLR was determined only at admission. This kind of methodological mistakes may contribute to the inconsistencies in the literature.

However, it seems that the CRP level is essential for identifying infections and cannot currently be replaced or improved by another parameter. Contrary to our expectations, neither ∆WBC count nor ∆NLR proved to be better diagnostic markers for infection. Combining the CRP level and NLR did not improve the detectability of infections either. We also examined whether NLR worked better than the CRP level in patients with different disease severities, but we did not find such an association. Accordingly, the best discriminatory cutoff level of both parameters increased in parallel with more severe AD stages.

During AD, NLR showed a good correlation, similar to other studies [7,8], with both 28-day [28] and 90-day mortality [27,29] and proved to be a more sensitive prognostic factor than the WBC count. Most studies have evaluated the NLR in patients with liver cirrhosis regardless of the presence or absence of an ongoing AD [12,30,31,32]. Those studies that investigated patients with AD, similarly to our present paper, typically only considered a specific subgroup, such as HBV-associated cirrhotic patients [33,34] or cirrhotic patients with TIPS [35]. Nonetheless, all studies found a significant association between mortality and NLR regardless of the exact population studied. On the other hand, ∆NLR did not have significantly higher prognostic value regarding mortality. The CRP level measured during AD showed a correlation with short-term mortality, but the area under the curve and odds ratio were smaller than those for NLR, suggesting that NLR might indicate more than just the extent of inflammation in this context. Considering the closer correlation of NLR with mortality than WBC or neutrophil count, it may be worth considering replacing the WBC count with NLR in AD score calculations. 

We have long been searching for a reliable method or molecule to early detect infections in patients with liver cirrhosis and distinguish them from noninfectious inflammations. However, there are two problems. First, during the initial stages of infections, the innate immune response dominates the host’s reaction. According to current knowledge, after the activation of pattern recognition molecules, the process follows a very similar pathway regardless of the response being triggered by DAMPs or PAMPs (inducing a similar set of response elements via activation of common, conserved early response pathways) [11,28,36,37]. The binding of DAMPs and PAMPs to pattern recognition molecules induces the release of inflammatory cytokines, acute-phase proteins, the formation of reactive oxygen and nitrogen species, the induction of antigen-presenting cells and, finally, the initiation of adaptive immune responses. Moreover, the presence of increasing intestinal bacterial translocation in parallel with the progression of liver cirrhosis causes pure sterile inflammation to be rare. While we can expect certain markers to increase significantly during manifest infections, distinguishing sterile from nonsterile inflammation will likely require identification at the level of DAMP–PAMP receptor binding. Therefore, our current parameters can at best be expected to show more significant changes in bacterial infections compared to nonpathogenic inflammations, but they are unlikely to allow for definitive differentiation. 

Our work has limitations. First, we recruited patients from a single center. Moreover, our cohort included individuals with both compensated and decompensated clinical stage before the AD episode. Furthermore, only data on the AD visit and the follow-up were collected in a prospective manner, while the baseline (non-AD visit) was collected retrospectively. Therefore, many patients did not have baseline CRP levels, and none of the patients had baseline PCT levels measured. Consequently, **∆**CRP and **∆**PCT values could not be calculated. Scheduling a baseline visit at a standard time before the AD episode was not possible for obvious reasons. This caused some variation in the time between the two events, although we included patients with a non-AD visit within one month before the AD episode. It is unknow whether the most optimal time window was selected. Including a serial of non-AD visits could address this question, with the potential to increase the diagnostic accuracy of delta values.

## 5. Conclusions

Based on the current results, it can be said that if the CRP level is above 15.5 g/mL and NLR is above 16.3 during AD, there is a high probability of the patient having a manifest bacterial infection. If NLR is below 2.2, the likelihood of infection is low. However, there remains a relatively wide gray zone where the clinician must make decisions based on the clinical picture. In acute decompensated patients, the NLR is associated with 28-day and 90-day mortality. These results seriously raise the question whether it would not be more appropriate to use the NLR instead of the WBC count to calculate the CLIF-C AD score.

## Figures and Tables

**Table 1:** Demographic, clinical and laboratory characteristics of patients with or without bacterial infections.

		Non-Iinfective (n = 107)	Infective (n = 126)	*p-*Value
Gender (male/female), n		74/33	71/55	0.057
Age (years) ×		56.3 ± 10.2	59.5 ± 9.8	**0.015**
Etiology #	Alcoholic	96 (89.7%)	101 (80.8%)	0.067
	Viral	15 (14.0%)	27 (21.6%)	0.171
	Autoimmune	5 (4.7%)	5 (4.0%)	1.000
	Other	0 (0.0%)	4 (3.2%)	0.126
CTP score (points) *		9 (7–10)	9 (8–10)	**0.041**
CTP stage #^	A	21 (19.6%)	21 (16.8%)	0.225
	B	54 (50.5%)	53 (42.4%)	
	C	32 (29.9%)	51 (40.8%)	
MELD score (points)		15 (12–20)	18 (14–24)	**0.004**
Comorbidities present #		64 (59.8%)	67 (53.2%)	0.354
HCC #		2 (1.9%)	14 (11.1%)	**0.007**
CLIF-C AD score (points)		46 (41–51)	49 (44–54)	**0.013**
CLIF-C AD sScore #	<50	61 (57.0%)	50 (39.7%)	0.076
	≥50	30 (28.0%)	40 (31.7%)	
ACLF #		16 (15.0%)	36 (28.6%)	**0.017**
ACLF grade #	1	11 (10.3%)	19 (15.1%)	0.543
	2	3 (2.8%)	9 (7.1%)	
	3	2 (1.9%)	8 (6.3%)	
WBC count * (10^9^/L)		7.15 (4.83–9.87)	8.35 (5.38–11.59)	0.072
ΔWBC count * (10^9^/L)		1.43 (−0.67–4.2)	3.04 (0.18–6.74)	**0.004**
Neutrophil count * (10^9^/L)		4.89 (3.24–7.65)	6.19 (3.59–9.38)	**0.020**
ΔNeutrophil count * (10^9^/L)		1.35 (−0.1–3.95)	2.91 (0.55–6.32)	**0.002**
Neutrophil percentage (%) *		69.6 (61–78.3)	76.55 (69.73–83.13)	**<0.001**
Lymphocyte count * (10^9^/L)		1.07 (0.79–1.63)	0.97 (0.61–1.61)	0.071
NLR *		4.02 (2.55–6.05)	6.13 (3.74–9.19)	**<0.001**
ΔNLR *		0.89 (0.07–3.13)	2.72 (1.08–6.51)	**<0.001**
CRP level * (mg/L)		10.09 (4.67–29.16)	32.89 (16.14–70.38)	**<0.001**
PCT level * (ng/mL)		0.18 (0.11–0.46)	0.43 (0.2–1.2)	**<0.001**

# n (%); × mean ± SD; * median (IQR); ^ CTP stage was not available for 1 patient; *p-*values were calculated with Mann–Whitney U-test, χ2-test or Fisher’s exact test as appropriate; *p-*values of <0.05 are statistically significant, bold letters indicate significant p values. HCC: hepatocellular carcinoma; MELD: mModel for end-stage liver disease score; CTP: Child–-Turcotte–-Pugh; CLIF-C AD score: CLIF Consortium aAcute dDecompensation score; ACLF: acute-on-chronic liver failure; WBC: white blood cell; NLR: neutrophil-to-lymphocyte ratio; CRP: C-reactive protein; PCT: procalcitonin; IQR: (inter quartile range: lowest 25%–highest 25%). Bold letters indicate significant *p* values.

**Table 2:** Baseline patient characteristics (non-AD).

Non-AD (Baseline)		
CTP score (points) *		8 (7–9)
CTP stage #^	A	108 (0.464)
	B	90 (0.386)
	C	4 (0.017)
MELD score (points)		11 (8–13)
WBC count *		5.31 (4.02–7.15)
Neutrophil count *		3.16 (2.54–4.5)
Neutrophil percentage (%) *		64.4 (56.95–69.7)
Lymphocyte count *		1.2 (0.81–1.7)
NLR *		2.76 (2.07–3.79)
CRP level *		4.67 (1.99–9.38)

# n (%); * median (IQR); ^ CTP stage was not available for 1 patient; MELD: model for end-stage liver disease score; CTP: Child–Turcotte–Pugh; WBC: white blood cell; NLR: neutrophil-to-lymphocyte ratio; CRP: C-reactive protein; IQR: inter quartile range, lowest 25%–highest 25%.

**Table 3:** Correlation between inflammatory parameters and Child–Turcotte–Pugh score or model for end-stage liver disease score at time of outpatient visit and acute decompensation.

Spearman’s Correlation		NLR	WBC Count (#)	Neutrophil Count (#)	Neutrophil Percentage (%)	Lymphocyte Count (n)	Lymphocyte Percentage (%)	CRP Level	
CTP score	r	0.12	0.025	0.028	0.086	−0.073	−0.141	0.217	Outpatient visit
	*p*	0.088	0.724	0.693	0.221	0.302	**0.046**	**0.004**	
MELD score	r	−0.019	−0.092	−0.116	−0.066	−0.077	−0.009	0.126	
	*p*	0.791	0.191	0.099	0.347	0.274	0.902	0.099	
CTP score	r	0.151	0.186	0.201	0.215	0.057	−0.122	0.214	AD visit
	*p*	**0.022**	**0.005**	**0.002**	**<0.001**	0.39	0.065	**0.001**	
MELD score	r	0.309	0.277	0.288	0.237	−0.045	−0.307	0.332	
	*p*	**<0.001**	**<0.001**	**<0.001**	**<0.001**	0.503	**<0.001**	**<0.001**	

r: correlation coefficient; CTP score: Child–Turcotte–Pugh score; MELD score: model for end-stage liver disease score; WBC: white blood cell; NLR: neutrophil-to-lymphocyte ratio; CRP: C-reactive protein; *p-*values of <0.05 are statistically significant; bold letters indicate significant *p*-values.

**Table 4:** ROC curve analysis and univariate Cox regression for predictors of infection.

	ROC Analysis			Univariable Logistic Regression		
	AUROC	95%CI	*p*	OR	95%CI	*p*
WBC count (10^9^/L)	0.568	0.495–0.642	0.072	1.074	1.015–1.136	**0.013**
ΔWBC count (10^9^/L)	0.610	0.538–0.682	**0.004**	1.111	1.041–1.187	**0.002**
Neutrophil count (10^9^/L)	0.589	0.516–0.661	**0.020**	1.106	1.036–1.181	**0.003**
Neutrophil percentage (%)	0.643	0.571–0.714	**<0.001**	1.043	1.017–1.069	**0.001**
NLR	0.660	0.59–0.73	**<0.001**	1.080	1.024–1.138	**0.004**
ΔNLR	0.662	0.592–0.733	**<0.001**	1.092	1.031–1.158	**0.003**
CRP level (mg/L)	0.736	0.673–0.8	**<0.001**	1.029	1.017–1.04	**<0.001**
PCT level (ng/mL)	0.688	0.604–0.772	**<0.001**	1.542	1.052–2.26	**0.027**

ROC: receiver operating characteristic; AUROC: area under the ROC curve; Cl: confidence interval; OR: odds ratio; WBC: white blood cell; NLR: neutrophil-to-lymphocyte ratio; ΔNLR: baseline NLR–NLR at acute decompensation; CRP: C-reactive protein, PCT: procalcitonin; *p-*values of <0.05 are statistically significant; bold letters indicate significant *p*-values.

**Table 5:** ROC curve analysis and univariate Cox regression for 28-day and 90-day mortality.

**28-Day** **Mortality**	**ROC Analysis**			**Univariable Cox Regression**		
	**AUROC**	**95%CI**	***p***	**HR**	**95%CI**	***p***
WBC count * (10^9^/L)	0.579	0.487–0.671	0.099	1.603	0.933–2.753	0.088
ΔWBC count * (10^9^/L)	0.601	0.511–0.69	**0.036**	1.269	0.939–1.713	0.121
Neutrophil count (10^9^/L) *	0.602	0.515–0.689	**0.033**	1.686	1.066–2.665	**0.025**
Neutrophil percentage (%)	0.689	0.612–0.766	**<0.001**	1.065	1.031–1.1	**<0.001**
NLR *	0.692	0.616–0.768	**<0.001**	1.772	1.386–2.264	**<0.001**
ΔNLR *	0.701	0.625–0.777	**<0.001**	1.481	1.199–1.83	**<0.001**
CRP level * (mg/L)	0.658	0.573–0.743	**0.001**	1.492	1.159–1.921	**0.002**
PCT level * (ng/mL)	0.748	0.656–0.84	**<0.001**	1.485	1.218–1.812	<0.001
**90-Day** **Mortality**	**ROC Analysis**			**Univariable Cox Regression**		
	**AUROC**	**95%CI**	***p***	**HR**	**95%CI**	***p***
WBC count * (10^9^/L)	0.583	0.498–0.668	0.057	1.741	1.082–2.801	**0.022**
ΔWBC count * (10^9^/L)	0.590	0.505–0.675	**0.039**	1.339	1.014–1.768	**0.04**
Neutrophil count (10^9^/L) *	0.610	0.529–0.691	**0.012**	1.806	1.209–2.698	**0.004**
Neutrophil percentage (%)	0.706	0.636–0.776	**<0.001**	1.068	1.038–1.098	**<0.001**
NLR *	0.702	0.63–0.773	**<0.001**	1.830	1.465–2.285	**<0.001**
ΔNLR *	0.693	0.618–0.767	**<0.001**	1.526	1.26–1.848	**<0.001**
CRP level * (mg/L)	0.662	0.585–0.739	**<0.001**	1.497	1.202–1.864	**<0.001**
PCT level * (ng/mL)	0.740	0.65–0.829	**<0.001**	1.476	1.24–1.758	**<0.001**

ROC: receiver operating characteristic; AUROC: area under the ROC curve; Cl: confidence interval; HR: hazard ratio; WBC: white blood cell; NLR: neutrophil-to-lymphocyte ratio; ΔNLR: baseline NLR–NLR at acute decompensation; CRP: C-reactive protein; PCT: procalcitonin; *p-*values of <0.05 are statistically significant; bold letters indicate significant *p*-values. * Logarithmically transformed variables.

**Table 6:** Univariable Cox regression of non-ACLF AD patients for 28-day and 90-day mortality.

Univariable Cox Regression of Non-ACLF AD Patients			
28-Day	HR	95%CI	*p*
WBC count (10^9^/L)	1.77	0.722–4.336	0.212
ΔWBC count (10^9^/L)	1.144	0.742–1.764	0.542
CRP level (mg/L)	1.239	0.852–1.802	0.263
PCT level (ng/mL)	1.602	1.051–2.441	**0.028**
NLR	1.958	1.093–3.508	**0.024**
ΔNLR	1.545	1.035–2.306	**0.033**
**90-Day**	**HR**	**95%CI**	***p***
WBC count (10^9^/L)	1.418	0.706–2.851	0.327
ΔWBC count (10^9^/L)	1.095	0.769–1.558	0.616
CRP level (mg/L)	1.243	0.926–1.668	0.148
PCT level (ng/mL)	1.403	0.972–2.025	0.071
NLR	1.856	1.176–2.928	**0.008**
ΔNLR	1.489	1.081–2.052	**0.015**

AD: acute decompensation; ACLF: acute-on-chronic liver failure; Cl: confidence interval; HR: hazard ratio; WBC: white blood cell; NLR: neutrophil-to-lymphocyte ratio; ΔNLR: baseline NLR–NLR at acute decompensation; CRP: C-reactive protein; PCT: procalcitonin; *p-*values of <0.05 are statistically significant; bold letters indicate significant *p*-values. All variables are logarithmically transformed.

## Data Availability

The data presented in this study are available on request from the corresponding author. The data are not publicly available due to Hungarian regulations on medical records.
